# Supplementary material for: Pollination treatment affects fruit set and modifies marketable and storable fruit quality of commercial apples
Source: R Soc Open Sci. 2019 Dec 11;6(12):190326. doi: 10.1098/rsos.190326 (PMC6936272; doi:10.1098/rsos.190326)
Supplement: Fruit set and interaction plots, and results for additional quality metrics [file rsos190326supp1.docx]

**Electronic supplementary material for the paper:**

**Pollination treatment affects fruit set and modifies marketable and storable fruit quality of commercial apples**

Ulrika Samnegård, Peter A. Hambäck & Henrik G. Smith

Corresponding author: Ulrika Samnegård, email: [ulrika.samnegard@biol.lu.se](mailto:ulrika.samnegard@biol.lu.se), [ulrika.samnegard@gmail.com](mailto:ulrika.samnegard@gmail.com)

**B**

**A**


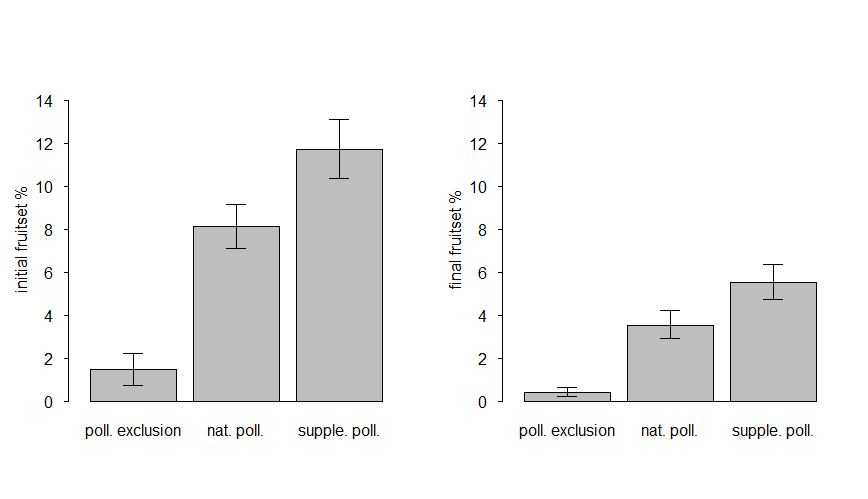


**Figure S1.** The effect of the treatments “pollinator exclusion”, “natural pollination” and “supplementary hand-pollination”, where the fruit set on the entire branch was considered for all treatments (compared to the main analyses where only flowers receiving the actual treatments were considered), on A) the initial fruit set (number of initial fruits divided by number of flowers per branch) and B) final fruit set (number of ripe fruits divided by number of flowers per branch), using predicted values from the GLS-models. Bars represent model-estimated standard errors. The initial fruit set was analysed with a generalized least square model (GLS) to allow for different variances between pollination treatments. Estimated means per treatment and the standard errors in B) are back-transformed from squared rooted values. In the supplementary hand-pollination treatment the fruit set was calculated for the entire branch but only 5-10 flowers on the branch was supplementary hand-pollinated. The results differ from when only supplementary hand-pollinated flowers were considered (Fig 1) by retaining the difference in fruit set between the supplementary hand pollinated and also for the final fruit (56% higher final fruit set for supplementary hand pollinated compared to natural pollination treatment, p =0.04).


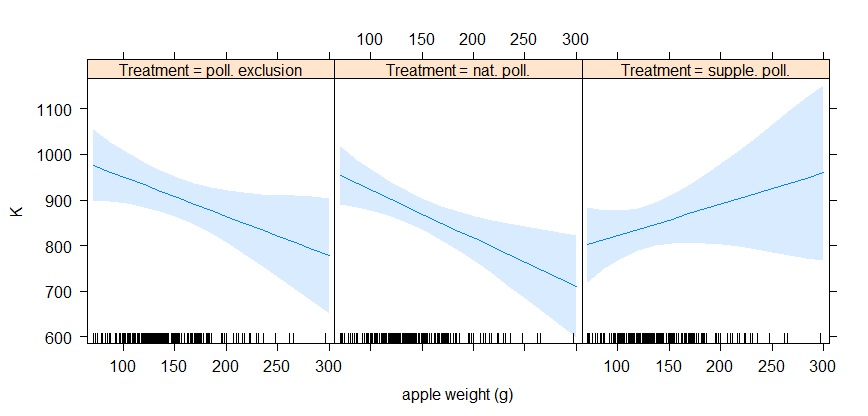


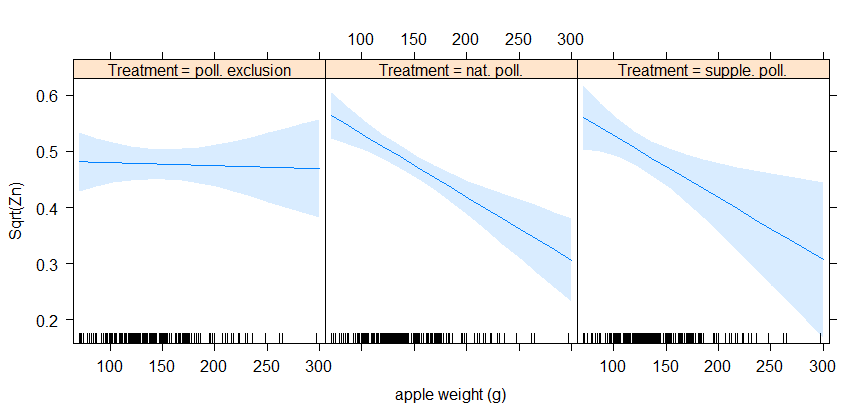


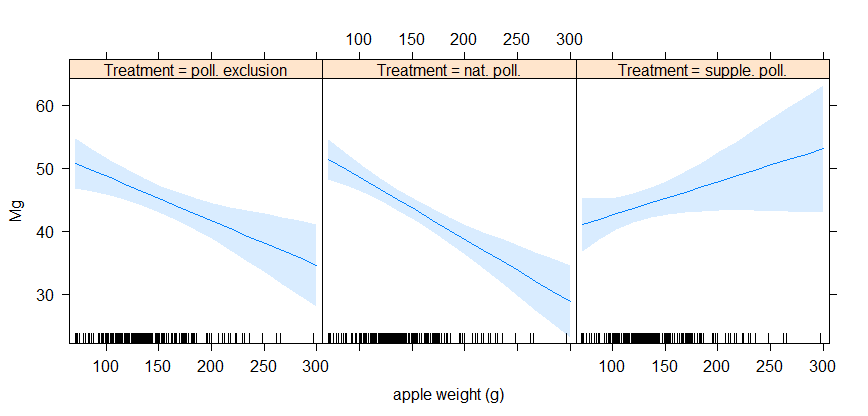


**Figure S2.** Interaction plots (based on data from the linear mixed effect models in table 1), for the elements that were affected by a significant interaction between pollination treatment (pollinator exclusion, natural and supplementary hand-pollination) and apple weight. The solid lines with their 95% confidence band illustrate the relationships between element concentration and apple weight for the different treatments separately. The R-package “effects” was used to produce the plots (Fox 2003).


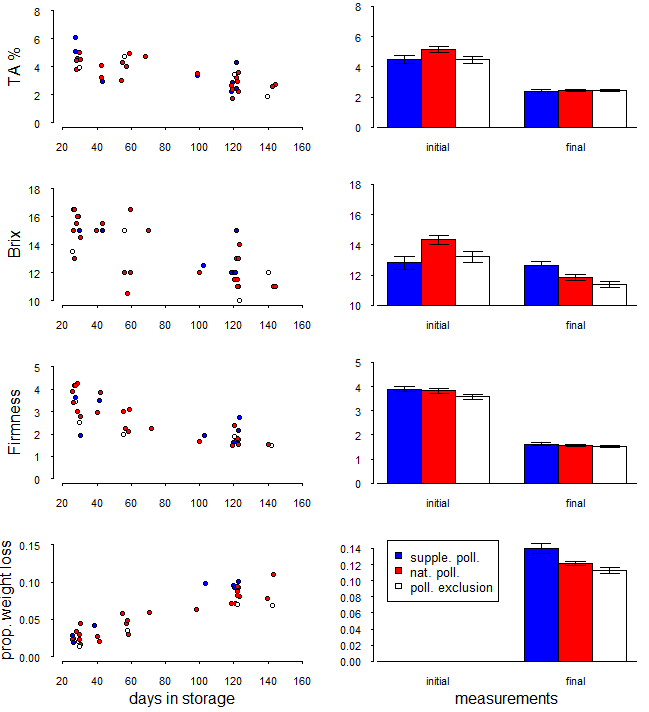


**A**

**B**

**Figure S3.** The quality measurements acidity (TA%), sugar (Brix), firmness and weight loss in relation to pollination treatment (supplementary hand-pollination, natural pollination and pollinator exclusion) and (A) storage time for apples that suffered from postharvest disorders, n =37, and (B) healthy fruit measured initially after harvest (initial) or in the end of the storage experiment (final), n = 153. Test statistics are presented in table S2.

**Table S1.** The wavelength used to analyses the different elements with the Inductively Coupled Plasma-Optical Emission Spectrometry and the average and standard deviation of the measured element concentrations (µg/g) in the apples in dry matter and fresh weight respectively. N = 214.

| Element | Wavelength | µg/g dry matter ± SD | µg/g fresh weight ± SD |
| --- | --- | --- | --- |
| K | 766.490 | 6471.8 ± 1596.5 | 899.4 ± 270.1 |
| P | 213.617 | 423.8 ± 138.5 | 59.0 ± 22.4 |
| Mg | 285.213 | 325.5 ± 76.5 | 44.7 ± 11.1 |
| Ca | 317.933 | 258.2 ± 106.6 | 34.8 ± 14.0 |
| B | 249.677 | 28.8 ± 11.2 | 3.9 ± 1.4 |
| Fe | 238.204 | 10.8 ± 9.7 | 1.5 ± 1.5 |
| Zn | 206.200 | 1.8 ± 0.7 | 0.24 ± 0.10 |

**Table S2.** Quality variables titratable acidity, brix, firmness and weight loss analysed against days in storage (for fruits that suffered from postharvest disorders) or storage category (for healthy fruit that where measured initially and in the end of the experiment), and pollinator treatment, fruit weight and site with linear mixed-effect models (lme, nlme-package in R) with treeID as random variable. Df = 2 for interaction and treatment effects and df = 1 for storage time, fruit weight and Site. P-values were obtained using log-likelihood ratio test.

|  | Log(TA%) | Brix | Log(Firmness) | Weight loss |
| --- | --- | --- | --- | --- |
|  |  |  |  |  |
| ***Fruits that suffered from post-harvest disorders*** |  |  |  |  |
| Treat*storage time (slope) | LR = 0.67  P = 0.71 | LR = 0.92  P = 0.63 | LR = 3.1  P = 0.21 | LR = 6.4  **P = 0.041**  Excl.: 0.0005 ± 0.0001  Cont.: 0.0006 ± 0.00005  Supp.: 0.0007 ± 0.00008 |
| Treat. | LR = 0.88  P = 0.64 | LR = 2.3  P = 0.32 | LR = 1.3  P = 0.52 |  |
| Storage time | LR = 14.4  **P < 0.001** | LR = 5.5  **P = 0.019** | LR = 38.1  **P < 0.001** |  |
| Fruit weight | LR = 0.33  P = 0.56 | LR = 1.5  P = 0.23 | LR = 19.9  **P < 0.001** | LR = 3.0  P = 0.085 |
| Site | LR = 2.4  P = 0.12 | LR = 35.5  **P < 0.001** | LR = 11.1  **P < 0.001** | LR = 0.05  P = 0.82 |
| ***Initial and final fruits*** |  |  |  |  |
| Treat*storage category (slope) | LR = 2.6  P = 0.27 | LR = 13.8  **P = 0.001**  Excl.: -1.40 ± 0.33  Cont.: -1.49 ± 0.29  Supp.: 0.24 ± 0.41 | LR = 0.74  P = 0.69 | - |
| Treat. | LR = 4.8  P = 0.092 |  | LR = 3.7  P = 0.10 | LR = 11.2  **P = 0.004**  Excl.: 0.18 ± 0.015  Cont.: 0.19 ± 0.014  Supp.: 0.20 ± 0.013 |
| Storage category | LR = 212.6  **P < 0.001** |  | LR = 399.4  **P < 0.001** | - |
| Fruit weight | LR = 0.15  P = 0.70 | LR = 0.06  P = 0.80 | LR = 20.1  **P < 0.001** | LR = 20.2  **P < 0.001** |
| Site | LR = 36.9  **P < 0.001** | LR = 46.1  **P < 0.001** | LR = 6.1  **P = 0.013** | LR = 4.5  **P = 0.034** |

## Table S3. The results, in terms of p-values, from the linear mixed-effects models (lme, nlme-package in R) analysing the relationship between the different elements as response variables and treatment, fruit weight, total buds per tree, final fruitset, colour cover and site as predictors. TreeID was included as a random effect in the models. P-values were obtained using log-likelihood ratio test. Bold p-values represent significant relationships (p < 0.05). Estimated R2 marginal (variation explained by fixed effects) and R2 conditional (variation explained by entire model) is Pseudo-R-squared for Generalized Mixed-Effect models. N = 188.

| Response variable | Treatment *  initial weight | Treatment | Initial weight | Total buds per tree | Final fruit set/branch | Colour cover | Site | Est. R^2^m | Est. R^2^c |
| --- | --- | --- | --- | --- | --- | --- | --- | --- | --- |
| Log(K_Ca) | 0.34 | 0.057 | 0.069 | 0.27 | **0.008** | 0.073 | **< 0.001** | 0.45 | 0.49 |
| Ca* | 0.20 | 0.10 | **0.002** | 0.98 | **0.010** | 0.45 | 0.63 | 0.12 | 0.15 |
| K | **0.025** |  |  | **0.035** | **0.014** | **0.001** | **< 0.001** | 0.75 | 0.84 |
| Sqrt(Zn) | **0.012** |  |  | 0.054 | 0.19 | 0.52 | **< 0.001** | 0.25 | 0.31 |
| Sqrt(P) | **0.062** |  |  | 0.23 | 0.95 | **0.008** | **< 0.001** | 0.72 | 0.78 |
| Log(Fe) | 0.30 | 0.14 | 0.99 | 0.72 | 0.55 | 0.26 | **0.014** | 0.11 | 0.11 |
| Mg | < **0.001** |  |  | 0.65 | 0.40 | **< 0.001** | **< 0.001** | 0.67 | 0.74 |
| Log(B) | 0.32 | 0.24 | 0.19 | **0.022** | 0.67 | 0.59 | 0.77 | 0.11 | 0.17 |

*two outliers were removed

## Table S4. Chi Squared likelihood ratio test statistics for all direct paths included in the final SEM. Bold p-values represent significant relationships (p < 0.05). The code used to produce the table was downloaded from: <https://github.com/jebyrnes/phrag_common_garden_sem> (Bowen *et al.* 2017).

| Response | Predictor | Chisq | Df | p-value |
| --- | --- | --- | --- | --- |
| Storage time | Log K:Ca ratio | 11.1 | 1 | < 0.001 |
|  | Site | 29.7 | 1 | < 0.001 |
|  | Fruit weight | 4.3 | 1 | 0.039 |
| Log K:Ca ratio | Poll. treatment | 3.6 | 2 | 0.17 |
|  | Fruit weight | 0.8 | 1 | 0.37 |
|  | Log final fruitset | 5.6 | 1 | 0.018 |
|  | Site | 39.7 | 1 | < 0.001 |
|  | Poll. treatment * Fruit weight | 7.6 | 2 | 0.022 |
| Fruit weight | Poll. treatment | 26.1 | 2 | < 0.001 |
|  | Log final fruitset | 4.4 | 1 | 0.035 |
|  | Site | 4.2 | 1 | 0.039 |
| Log final fruitset | Poll. treatment | 40.4 | 2 | < 0.001 |

**References**

Bowen, J.L., Kearns, P.J., Byrnes, J.E.K., Wigginton, S., Allen, W.J., Greenwood, M.*, et al.* (2017). Lineage overwhelms environmental conditions in determining rhizosphere bacterial community structure in a cosmopolitan invasive plant. *Nature Communications*, 8: 433.

Fox, J. (2003). Effect Displays in {R} for Generalised Linear Models. *Journal of Statistical Software*, 8: 1-27.
